# Supplementary material for: A web-database of mammalian morphology and a reanalysis of placental phylogeny
Source: BMC Evol Biol. 2007 Jul 3;7:108. doi: 10.1186/1471-2148-7-108 (PMC1941728; doi:10.1186/1471-2148-7-108)
Supplement: Additional File 1 — Website data: The 196 characters first described in [17], along with the supplementary data indicated below, have been joined into a number of HTML files, all of which are linked to the "morphsite_bmc07.html" index page. The embedded links to the supporting files are functional, independent of PC or platform, provided that the directory structure remains intact (i.e., all files remain in a single folder, no subdirectories). Nexus files: Datafiles containing the morphological, indel, and aligned DNA sequence data, linked from the "morphsite_bmc07.html" index page. Table 1. Changes to the multiple sequence alignment of [9]. Table 2. Qualitative character-state corrections to the morphology matrix of [17]. Table 3. Summary of discrepancies between Appendices 1 and 2 of [17]. [file 1471-2148-7-108-S1.zip › asher-BMC07-website/morphsite_bmc07.html]

asher-characterBank


|  |  |  |  |
| --- | --- | --- | --- |
| Morphological Image Database    Asher, 2007. A database of morphological characters and a combined-data reanalysis of placental mammal phylogeny.  BMC Evolutionary Biology   7: 108      Click here for a bibliography of citations among the character explanations & images    The images below are links to text- and graphics-rich explanations of a morphological dataset | Additional file 1:  - supplementary tables 1-3 (changes to multiple sequence alignment, changes to morphological character-states, corrections to appendices 1 and 2 of Asher et al. 2003).   - Combined data nexus file used for phylogeny reconstruction.  - Morphology nexus file including names of characters and states.  - Sequence alignment nexus file based on Roca et al. (2004) corresponding to changes given in table 1 of Additional  File 1 (above). | Images on this website are derived primarily from the collections of several natural history museums: | This work is licensed under a    Creative Commons License.   Please cite Asher RJ. 2007. BMC Evolutionary Biology 7:108 if images/text from this site are used elsewhere.    Corrections/suggestions re: this website are welcome via contact info from the BMC paper or the author's website     Supported in part by the German Science Foundation (DFG) |
| Cranium | Dentition and Mandible | Postcranium | Soft Tissue |
